# Japan’s New Framework on Dementia Care

**DOI:** 10.1093/geroni/igab046.1487

**Published:** 2021-12-17

**Authors:** Kenji Toba

**Affiliations:** Tokyo Metropolitan Institute for Geriatrics and Gerontology , Tokyo Metropolitan Institute for Gerontology, Tokyo, Japan

## Abstract

The number of people with dementia in Japan is ever-increasing. In 2020, 6 million people lived with dementia. The number is expected to increase to 9 million in 2040. This means that a person with dementia will be supported by three working people. To prepare for the big wave of dementia, Japan released the New Orange Plan in January 2015. In 2019, the Framework for Promoting Dementia Care was issued by the Japanese cabinet in which prevention and the opportunity for persons with dementia to age in place were set as the main goals. This framework requires all ministries to promote people’s awareness about individuals with cognitive impairment. The educational targets include taxi drivers, retail shop clerks, bankers, police, and people working in the criminal justice system. I will discuss the New Framework which has potential to assist the country in supporting people living with dementia.

